# Insights into the Mechanisms of Action of MDA-7/IL-24: A Ubiquitous Cancer-Suppressing Protein

**DOI:** 10.3390/ijms23010072

**Published:** 2021-12-22

**Authors:** Jinkal Modi, Abhishek Roy, Anjan K. Pradhan, Amit Kumar, Sarmistha Talukdar, Praveen Bhoopathi, Santanu Maji, Padmanabhan Mannangatti, Daniel Sanchez De La Rosa, Jiong Li, Chunqing Guo, Mark A. Subler, Jolene J. Windle, Webster K. Cavenee, Devanand Sarkar, Xiang-Yang Wang, Swadesh K. Das, Luni Emdad, Paul B. Fisher

**Affiliations:** 1 Department of Human and Molecular Genetics, Virginia Commonwealth University, Richmond, VA 23218, USA; modij2@mymail.vcu.edu (J.M.); abhishek.roy@vcuhealth.org (A.R.); anjan.pradhan@vcuhealth.org (A.K.P.); amit.kumar@vcuhealth.org (A.K.); sarmistha.talukdar@vcuhealth.org (S.T.); praveen.bhoopathi@vcuhealth.org (P.B.); santanu.maji@vcuhealth.org (S.M.); padmanabhan.mannangatti@vcuhealth.org (P.M.); daniel.sanchezdelarosa@vcuhealth.org (D.S.D.L.R.); chunqing.guo@vcuhealth.org (C.G.); mark.subler@vcuhealth.org (M.A.S.); jolene.windle@vcuhealth.org (J.J.W.); devanand.sarkar@vcuhealth.org (D.S.); xiang-yang.wang@vcuhealth.org (X.-Y.W.); swadesh.das@vcuhealth.org (S.K.D.); 2 VCU Institute of Molecular Medicine, Virginia Commonwealth University, Richmond, VA 23218, USA; 3 Department of Medicinal Chemistry, School of Pharmacy, Virginia Commonwealth University, Richmond, VA 23218, USA; jli29@vcu.edu; 4 VCU Massey Cancer Center, Virginia Commonwealth University School of Medicine, Richmond, VA 23218, USA; 5 Ludwig Institute for Cancer Research, University of California San Diego, La Jolla, CA 92037, USA; wcavenee@ucsd.edu

**Keywords:** MDA-7/IL-24, cytokine, apoptosis, bystander antitumor activity, combinatorial therapy

## Abstract

Melanoma differentiation associated gene-7/interleukin-24 (MDA-7/IL-24), a secreted protein of the IL-10 family, was first identified more than two decades ago as a novel gene differentially expressed in terminally differentiating human metastatic melanoma cells. MDA-7/IL-24 functions as a potent tumor suppressor exerting a diverse array of functions including the inhibition of tumor growth, invasion, angiogenesis, and metastasis, and induction of potent “bystander” antitumor activity and synergy with conventional cancer therapeutics. MDA-7/IL-24 induces cancer-specific cell death through apoptosis or toxic autophagy, which was initially established in vitro and in preclinical animal models in vivo and later in a Phase I clinical trial in patients with advanced cancers. This review summarizes the history and our current understanding of the molecular/biological mechanisms of MDA-7/IL-24 action rendering it a potent cancer suppressor.

## 1. Introduction

**Figure 1:**
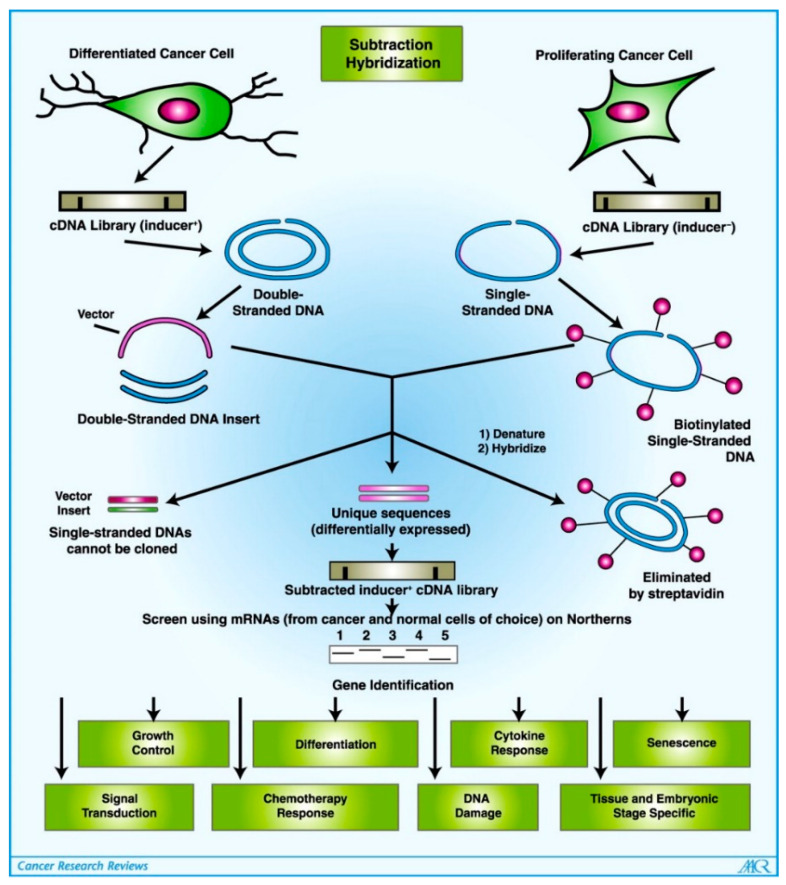
**Subtraction hybridization identifies MDA-7/IL-24**. Schematic of DISH (differentiation induction subtraction hybridization), an approach for identifying and cloning genes associated with the induction of terminal differentiation in human melanoma cells. Treatment of HO-1 human melanoma cells with a combination of IFN-β + mezerein results in a rapid and irreversible loss of proliferation, extinction of tumorigenic potential, and terminal differentiation [12,13,14,15,16]. The DISH approach was developed to identify and clone genes associated with and causative of the physiologic changes associated with terminal differentiation. mRNAs were isolated from actively proliferating and IFN-β + mezerein (2000 units/mL + 10 ng/mL)-treated HO-1 cells that span the first 24 h of treatment, and were converted into cDNAs. Subtraction hybridization was then done between differentiation inducer-treated and control-proliferating cancer cells resulting in the production of a subtracted cDNA library enriched for melanoma differentiation associated (*mda*) genes. Probing of clones isolated from this cDNA library permitted cloning of *mda* genes involved in critical cellular processes, some are listed here [12,13,14,15,16]. Reprinted by permission from Fisher, 2005 [17].

Melanoma differentiation associated gene-7/Interleukin-24 (MDA-7/IL-24) is a unique cytokine belonging to the IL-10 gene family that was cloned using subtraction hybridization in the early-nineties [1,2] (Figure 1). Based on a conserved stretch of amino acids, chromosomal location and cytokine-like properties, MDA-7/IL-24 was considered a member of the IL-10 gene family which consists of IL-19, IL-20, IL-22, and IL-26 [3]. Protein expression of MDA-7/IL-24 is decreased during progression from melanocyte to melanoma and remains undetectable in metastatic melanoma lending to its classification as a putative tumor suppressor. The research completed previously has shown that ectopic expression of *mda-7/IL-24* using transfection of tumor cells with plasmid cDNA or adenovirus-mediated delivery, or exposure to a purified protein results in the suppression of tumor cell growth [1,2,4,5,6,7]. Although our understanding of the molecular actions of *mda-7/IL-24* have been clarified over the years, we still have much to learn [6,7]. Initial studies in a Phase I clinical trial in patients with advanced cancers confirmed that intratumoral injection of an adenovirus (Ad) expressing *mda-7/IL-24* (Ad.*mda-7*; INGN 241) is safe and clinically effective in inducing cancer cell-specific apoptosis. Future studies using diverse ways of administering *mda-7/IL-24* as a single agent, including viral administration [8,9,10] and T cell mediated delivery [11], and in combination with other therapeutic agents are planned for the near future and suggest that this therapeutic cytokine will provide significant clinical benefit in patients with diverse cancers.

The expression profile of *mda-7/IL-24* suggests a versatile gene whose normal physiological functions are associated with certain aspects of immunoregulation. The adenovirus-mediated delivery of *mda-7* (Ad.*mda-7*) selectively inhibits growth and induces apoptosis and toxic autophagy in a wide-range of human cancer cells without inducing harmful effects in normal cells [6,7,16,17,18,19,20,21]. In this review, we review specific aspects of MDA-7/IL-24 function including its anti-cancer properties, combinatorial effects with other agents, early Phase I clinical trial and future prospects.

## 2. Identification and Structure of MDA-7/IL-24

*mda-7* was first identified in a human melanoma cell line (HO-1) using a subtraction hybridization technique applied to HO-1 metastatic human melanoma cells irreversibly stimulated to lose their capability of cell division and terminally differentiate after the combination treatment with IFN-ß plus Mezerein (Protein Kinase C activator) [1,2]. This technique identified the *mda-7* cDNA from temporal cDNA libraries produced from IFN-ß + MEZ-treated HO-1 cells after subtracting the temporal cDNA libraries of actively proliferating HO-1 melanoma cells. This distinctive cDNA encodes a unique protein of 206 amino acids with a 23.8 kDa molecular weight. Later, Wang et al. reported that MDA-7/IL-24 acts as potent ligand for two heterodimeric receptors IL-20R1/IL-20R2 and IL-22R1/IL-20R2 [22]. Subsequent studies by our research team demonstrated that MDA-7/IL-24 can also signal through the IL-22R2/IL-21R1 receptors [23].

MDA-7/IL-24 was found to be evolutionarily conserved among species. Southern blot analysis revealed that the orthologous sequences of the human *mda-7* gene can be identified in murine, canine, feline, simian, bovine, and yeast genomes [2,5,6]. In a rat wound healing model, Soo et al. identified and cloned a rat ortholog of MDA-7/IL-24, the expression of which was elevated in fibroblast-like cells during the inflammatory and granulation phase of wound healing [24]. In 2000, Zhang et al. cloned Rat IL-24 (MOB-5) by differential display as a differentially expressed gene induced by oncogenic Ha-Ras in non-transformed Rat-1 cells [25]. MOB-5 encodes a cytokine-like secreted protein, shares sequence identity at both the DNA and a protein level to human MDA-7/IL-24 [26]. Human MDA-7/IL-24 and rat C49A/MOB-5 only share ∼58.7% homology in their amino acid sequences and display diverse growth-related functions, which suggest that they may be related molecules, rather than actual homologues [18]. Schaefer et al. identified a murine ortholog of human *mda-7*, which is known as FISP (Interleukin 4-induced secreted protein) [27]. FISP is shown to be expressed in Th2 cells during their differentiation, exhibits 93% and 69% amino acid similarity to rat c49a and human MDA-7, respectively, and possesses antitumor properties [18,27]. Sandey and colleagues studied the genomic structure and expression profile of the canine ortholog of the human *mda-7/IL-24* gene and found that the *mda-7* locus is evolutionarily conserved in dogs, but it has a more restrictive pattern of tissue expression than in humans [28]. Five splice variants of canine *mda-7* were reported that encode four protein isoforms of the canine MDA-7. Similar to human MDA-7/IL-24, canine MDA-7 has a potential signal peptide and conserved IL-10 signature motif [28]. Due to the high amino acid sequence similarity with human MDA-7/IL-24, canine MDA-7 is also predicted to have similar antitumor properties which currently are under investigation. In summary, although mouse (FISP), rat (MOB-5) and canine MDA-7 display wide-ranging sequence homologies with human MDA-7/IL-24 the functions of these proteins vary between species [18,19,20,22,24,25,26,27,28].

The human IL-24 gene contains 6 introns and 7 exons, and is located within the 1q32-33 chromosome in the cluster region of IL-19 and IL-20 cytokine genes [29,30,31]. Nearly 24–33% amino acid sequence homology has also been reported with other IL-20 cytokine family members [22]. Interestingly, despite its long history of interesting and novel functions, the structural details have only recently been revealed by determining the crystal structure of the ternary complex of IL-24 and its receptors IL-22R1 and IL-20R2 at 2.15Å resolution [32]. Lubkowski et al. also reported on the low stability of the IL-24 protein due to lack of disulfide structural restraint [32]. This study also clarified the higher affinity of IL-20R2 than IL-22R1 receptor for the MDA-7/IL-24 cytokine [32]. Notwithstanding the structural details, studies suggest that although, by sequence similarity, IL-24 belongs to the subfamily of IL-10, its specific arrangement of disulfide bonds and unique surface properties renders it a special identity as a one of its kind unique antitumor cytokine. Its cytokine activity, secretion and stability are partially dependent upon its post-translational modifications including glycosylation (for solubility and bioavailability) and disulfide bonds (for secretion from host cells) [33].

## 3. Transcriptional Regulation of MDA-7/IL-24

The gene promoter region of the *mda-7/IL-24* gene was initially characterized in HO-1 cells in the Fisher laboratory, and upon IFN-ß + Mezerein treatment, its expression was dependent upon C/EBP-β (member of CEBP family) and c-Jun (member of AP-1 family) transcription factors. These transcriptional regulators directly bind to the MDA-7/IL-24 promoter to elevate gene expression [34]. The mechanism underlying a gradual decrease in expression during melanoma progression involves AU rich 3′ UTR elements (ARE) in the MDA-7/IL-24 transcript, which renders the MDA-7/IL-24 mRNA very unstable. This loss of mRNA stability can be rescued by IFN-ß + Mezerein treatment, resulting in the expression of *mda-7/IL-24* [35]. Although the principal factor regulating the expression of *mda-7/IL-24* is its mRNA stability, a major pathway reported to be involved in the induction of *mda-7/IL-24* expression due to IL-1ß treatment in keratinocytes is the p38 MAPK signaling pathway. Furthermore, inhibition of this pathway leads to decreased MDA-7/IL-24 protein expression due to the destabilization of *mda-7/IL-24* mRNA [36]. MDA-7/IL-24 expression was also reported at elevated levels in the inflamed mucosal tissue of inflammatory bowel disease patients and similar regulatory mechanisms, i.e., AP-1 and CEBP, mediated activation of the *mda-7/IL-24* promoter region due to IL-1ß stimulation in human colonic subepithelial myofibroblast (SEMF) cells [37].

**Figure 2:**
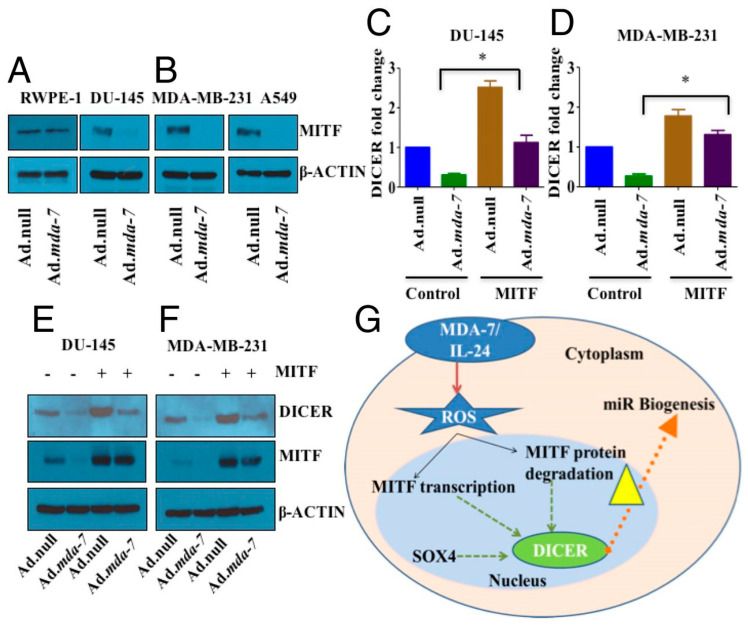
***mda-7/IL-24*****-mediated DICER regulation is controlled by the transcription factor MITF**. *mda-7/IL-24* downregulates MITF in different cancer cell lines (**A**,**B**), but not in normal RWPE-1 cells (**A**). DU-145 (**C**) and MDA-MB-231 cells (**D**) were transfected with vector or MITF, and then treated with Ad.null or Ad.*mda-7*. RNA was isolated 72 h post infection, and real-time quantitative PCR was done to check the level of DICER. DU-145 (**E**) and MDA-MB-231 cells (**F**) were treated as described in (**C**,**D**), total protein was isolated, and Western blotting was done with DICER and MITF antibodies. Actin was used as a loading control. (**G**) Schematic representation of regulation of the miRNA processing enzyme DICER by *mda-7/IL-24*. MDA-7/IL-24 downregulates the transcription factor MITF in a ROS-dependent manner, which in turn downregulates DICER. RWPE-1 (immortalized human prostate epithelial cell line); DU-145 (human prostate cancer cell line); MDA-MB-231 (human breast cancer cell line); and A549 (human lung cancer cell line). Reprinted by permission from Pradhan et al., 2019 [39]. *: *p* < 0.05.

Epigenetic processes are important modulators of the transcriptional regulation of tumor promoting and tumor suppressor genes, including *mda-7/IL-24* transcriptional control. In human melanoma (A375) cells, the HDAC inhibitors, sodium butyrate and Trichostatin A (TSA), upregulate MDA-7/IL-24 expression and downregulation is observed upon overexpression of the HDAC4 enzyme [38]. Recently, our group has shown that MDA-7/IL-24 delivery, either by means of a type 5 adenovirus (Ad.5-*mda-7*) or with purified MDA-7/IL-24 protein, inhibits the DICER regulatory mechanism that is essential for microRNA (miRNA) processing in cells [39]. miRNAs are small noncoding RNAs that function as major players of posttranscriptional gene regulation in diverse species. In mammals, the biogenesis of miRNAs is executed by cooperation of multiple biochemical reactions including processing of miRNA precursors by two central endoribonucleases, Drosha and Dicer [40]. These studies suggest a unique relationship between *mda-7/IL-24* and miRNA production The process of DICER downregulation was specific for only mature miRNA-221 (not for pri-miRNA-221) and this effect was very specific against all cancer cells, without effecting normal prostate epithelial cells (Figure 2). Additionally, no adverse effects or changes in miRNA were observed relative to other miRNA processing cofactors, including Argonaute or DROSHA [39]. Several previous reports also establish the use of MDA-7/ IL-24 and epigenetic therapeutics as a combination treatment against cancer [41,42,43]. An Ad expressing both MDA-7/IL-24 and miRNA-34a under an endogenous E3 promoter displayed higher antitumor activity than the individual therapeutic activity of these molecules when used alone in hepatocarcinoma (HCC) cells [44].

## 4. Tumor Suppressor Role of MDA-7/IL-24

### 4.1. Apoptosis and Autophagy

**Figure 3:**
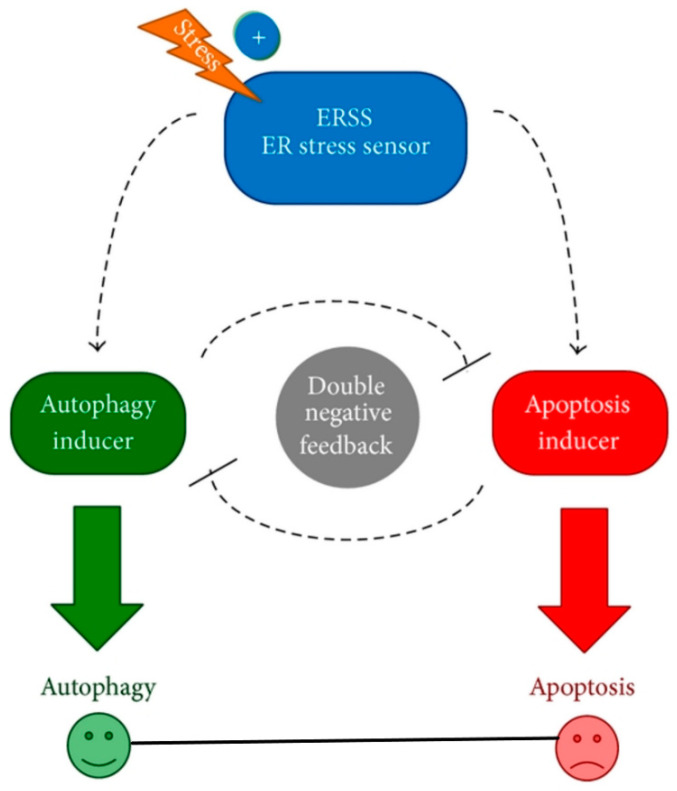
**The relationship between apoptosis and autophagy**. The schematic model of autophagy-apoptosis crosstalk during ER stress. The autophagy inducer, the apoptosis inducer, and the ER stress sensor (ERSS) are denoted by isolated green, red, and blue boxes, respectively. Dashed line shows how the molecules can influence each other, while blocked end lines denote inhibition. Reprinted by permission from Holczer et al., 2015 [46].

The tissues of multicellular organisms consist of multiple cell types that are organized into a highly organized hierarchy and complex structure. The regulation of this hierarchy is not only regulated by cell-division, but also through cell death. The process of programmed cell death, also known as apoptosis, occurs due to activation of an intracellular death program removing non-essential and damaged cells (Figure 3). Autophagy is a self-regulatory process that is important for balancing sources of energy in evolving situations such as stress (nutrient, hypoxia) [45]. A group of intracellular proteases called caspases are present as inactive pro-enzymes, activated by proteolytic cleavage.

The mitochondria are central communication command centers for both caspase-dependent and caspase-independent death pathways. Mitochondria respond to multiple death stimuli including those in which pro-apoptotic Bcl2 family proteins, such as Bax/Bak, induce mitochondrial membrane permeabilization and cause the release of apoptotic molecules [47,48,49,50,51]. Multiple death stimuli with or without the involvement of the classical Bcl family proteins converge on the mitochondria to trigger the release of pro-apoptotic molecules to initiate the death cascade [47,50,52].

**Figure 4:**
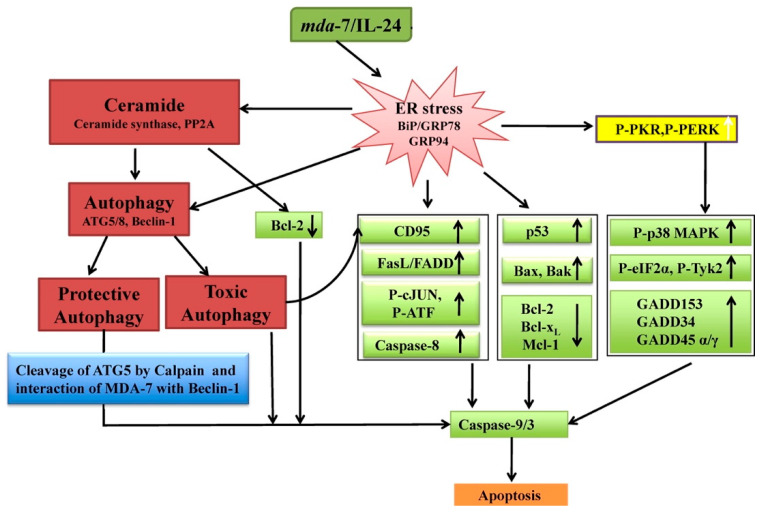
**Model of *mda-7/IL-24*-induced apoptosis in cancer cells**. Outline of proposed selective cytotoxic effects of *mda-7/IL-24* on cancer versus normal cells. When ectopically overexpressed, MDA-7/IL-24 localizes in the ER/Golgi compartments, regardless of the presence or absence of a secretory signal in the protein. Accumulation of MDA-7/IL-24 protein in transformed/tumor cells in this compartment triggers apoptosis, toxic autophagy and induction of ceramide that could involve induction of ER stress and/or reactive oxygen species in mitochondria. MDA-7/IL-24 activates signal transduction pathways and/or potentially enters cancer cells and activates pro-apoptotic pathways by localization and accumulation in the ER/Golgi compartment and/or by inducing mitochondrial dysfunction. A combination of pathways triggered by *mda-7/IL-24* results in transformed cell-specific apoptosis (or toxic autophagy). Adapted by permission from Dash et al., 2010 [55].

**Figure 5:**
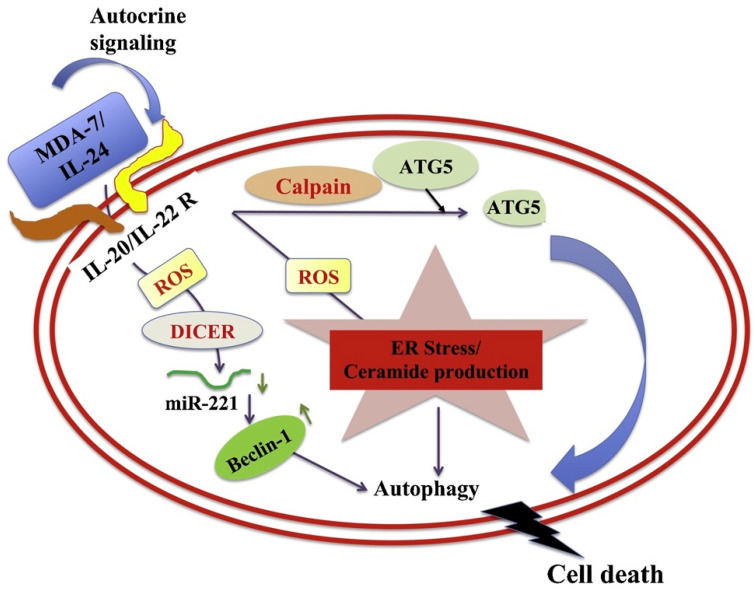
**A schematic representation of how MDA-7/IL-24 regulates toxic autophagy in cancer cells leading to cell death.** MDA-7/IL-24 regulates autophagy mediated through ER stress and ceramide production. MDA-7/IL-24 first interacts with its receptors which leads to a downstream signaling pathway mediated by reactive oxygen species (ROS). ROS regulates DICER and through this molecule MDA-7/IL-24 downregulates miR-221, which in turn upregulates Beclin-1 to induce toxic autophagy leading to cell death. The transition of protective to toxic autophagy is explained by the cleavage of ATG5 by Calpain, which is also mediated by ROS induced from MDA-7/IL-24 treatment. Reprinted by permission from Emdad et al., 2020 [7].

MDA-7/IL-24 regulates mitochondrial apoptotic pathways and oxidative stress (Figure 4) [7,17,18,19,20,53,54,55]. Direct anti-cancer effects are evident when MDA-7/IL-24 is overexpressed in cancerous cells [4,5,6,7,17,18,19,20,55]. MDA-7/IL-24 overexpression leads to up-regulation of pro-apoptotic genes such as Bax, Bad and others and downregulation of anti-apoptotic genes such as Bcl-xL and Bcl-2 in cancer cells. This alteration in expression levels of pro-apoptotic genes and anti-apoptotic genes tilts the balance toward cell death. MDA-7/IL-24 induces cell-death/apoptosis by promoting endoplasmic reticulum (ER) stress-induced apoptosis and toxic autophagy (Figure 4) [6,7,16,17,18,19,20,21,55]. Induction of ceramide production contributes to ER stress-induced apoptosis in cancer cells [56,57,58]. The interaction of MDA-7/IL-24 occurs with its receptors IL-20/IL-22, leading to the activation of downstream signaling cascades operated by reactive oxygen species (ROS) (Figure 5). The modulation by ROS further regulates the miRNA processing enzyme DICER. The subclass of miRNAs, miRNA-221 is considered to be downregulated by MDA-7/IL-24 which activates transcriptional target Beclin-1 [59,60]. The upregulation of Beclin-1 induces toxic autophagy leading to apoptotic death (Figure 5) [59].

### 4.2. Anti-Angiogenesis

Angiogenesis is the process of new growth of blood vessels from the existing vasculature. It occurs throughout life in both health and disease, beginning in utero and continuing through old age. No metabolically active tissue in the body is more than a few hundred micrometers from a blood capillary, which is formed by the process of angiogenesis. Capillaries are needed in all tissues for diffusion exchange of nutrients and metabolites. Changes in metabolic activity lead to proportional changes in angiogenesis and, hence, proportional changes in the number and distribution of capillaries. Oxygen plays a pivotal role in this regulation. Angiogenesis critically regulates the growth of cancer because solid tumors need a blood supply if they are to grow beyond a few millimeters in size. Tumors can actually cause this blood supply to increase by secreting chemical signal molecules that stimulate angiogenesis [61]. Tumors express molecules such as vascular endothelial growth factor (VEGF), a signaling protein that promotes the growth of new blood vessels and interleukin-8 (IL-8), which facilitates the production of tumor blood vessels [62]. The regulation of tumor angiogenesis occurs through a regulated balance between angiogenic and anti-angiogenic processes. Many clinical studies have tested various anti-angiogenic drugs designed to inhibit tumor growth by blocking tumor angiogenesis. The regulation of angiogenesis in a tumor is defined at multiple levels, either by obstructing new vessel formation or blocking the vessel itself.

MDA-7/IL-24′s anti-angiogenic effect is evident in human umbilical vascular endothelial cells, also known as HUVEC [63]. Infection of HUVEC cells with Ad.*mda-7* inhibits endothelial cell differentiation [63,64]. MDA-7/IL-24 protein secreted from Ad.5-*mda-7* infected HEK293 cells has potent anti-angiogenic activity and can inhibit the differentiation of endothelial cells more potently than endostatin [63]. This cytokine also inhibits the migratory potential of endothelial cells [63]. Experimental evidence suggests that MDA-7/IL-24 treatment inhibits blood vessel formation in a dose-dependent manner in vitro [65]. Similarly, treatment of tumor xenografts with MDA-7/IL-24 reduces expression of angiogenesis markers [66]. MDA-7/IL-24 protein results in the inhibition of pro-angiogenic factors such as VEGF and basic fibroblast growth factor [67]. The PI3K/Akt signaling cascade plays a seminal role in the regulation of angiogenesis, and the downregulation of phosphor-Akt by MDA-7/IL-24 can therefore negatively modulate angiogenesis [63,65]. Combined treatment of mda-7/IL-24 gene therapy with radiotherapy resulted in better outcomes in NSCLC than only radiotherapy [67]. The inhibitory effect of MDA-7/IL-24 on VEGF regulation is mediated by Src kinase [68]. A study by Inoue et al. revealed that MDA-7/IL-24 inhibits c-Src kinase activity and abrogates STAT-3 binding to the VEGF promoter, which ultimately results in a decrease in VEGF mRNA and protein levels [68].

### 4.3. Anti-Metastasis and Anti-Invasion

**Figure 6:**
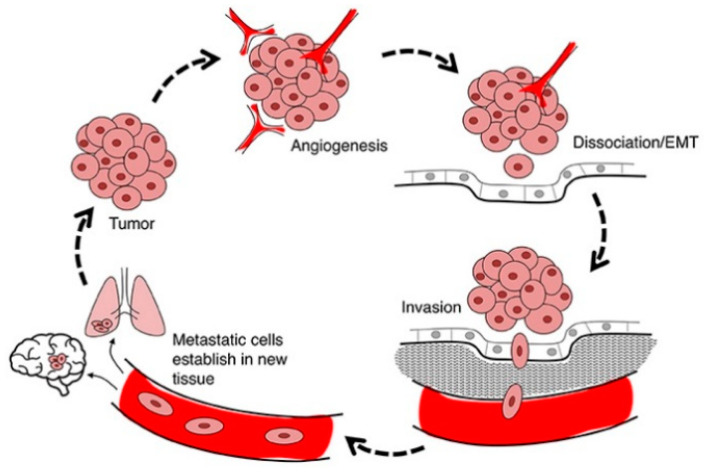
**Invasion and Metastasis**. Clinically detectable metastases represent the end products of a complex series of cell-biological events, which are collectively termed the invasion-metastasis cascade. During metastatic progression, tumor cells exit their primary sites of growth (local invasion, intravasation), translocate systemically (survival in the circulation, arrest at a distant organ site, extravasation), and adapt to survive and thrive in the foreign microenvironments of distant tissues (micro metastasis formation, metastatic colonization). Reprinted by permission from De Ieso and Yool, 2018 [74].

Cell migration is a pivotal component of cell invasion where motile cells can navigate through the extracellular matrix within a tissue or infiltrate into adjacent tissues. Cancer cells that become invasive may disseminate to secondary sites and form metastases (Figure 6). There is evidence that *mda-7/IL-24* blocks the migration and invasion of cancerous cells [69]. This anti-cancer outcome directly correlates with downregulation of various signaling cascades such as expression of PI3K (phosphatidylinositol 3-kinase), FAK (focal adhesion kinase), and MMP-2 and MMP-9 (matrix metalloproteinase-2 and 9) [69]. *mda-7/IL-24* induces potent anti-invasion and anti-metastasis activities in cancers of the cervix, lung, liver and prostate [69,70,71,72,73]. The pathways involved in regulating invasion and metastasis include both receptor-independent as well as receptor-dependent processes [19].

### 4.4. Bystander Effect

An important component of *mda-7/IL-24′s* broad-spectrum antitumor activity involves cytokine secretion that mediates “bystander” anticancer activity [6,7,17,18,19,20,21,33,55,65]. This activity is displayed by both normal and cancer cells, while only the cancer cells die when *mda-7/IL-24* is expressed [75]. The process is initiated through secreted MDA-7/IL-24 protein interacting with dimeric IL-20/IL-22 receptors and operates through an autocrine/paracrine loop [75,76,77]. With very few exceptions, most normal and cancer cells contain either IL-20R1, IL-20R2 and/or IL-22R1 receptors, which can form dimeric receptor complexes, allowing them to respond to exogenous MDA-7/IL-24 resulting in autocrine induction of MDA-7/IL-24 production, the autocrine/paracrine loop [76]. The concept of “bystander” antitumor effect of MDA-7/IL-24 was first observed in pancreatic carcinoma cells [78,79] and later shown to be the result of an autocrine/paracrine loop [76]. Although, *mda-7/IL-24* mRNA is actively expressed in a wide array of human cancers when infected with a replication incompetent Ad expressing *mda-7/IL-24* (Ad.*mda-7*), pancreatic cancer cells display a unique resistance phenotype involving the inability to translate *mda-7/IL-24* mRNA into protein [78,79]. After infection of human pancreatic cancer cells with Ad.*mda-7*, at doses effective in other cancers, growth was not suppressed, and apoptosis was not induced. Pancreatic cancer is a complex disease without any currently effective therapy [80]. A predominant genetic change in 85 to 95% of pancreatic cancers is activation of the K-Ras oncogene [81]. When K-Ras expression was abolished using pharmacological or genetic approaches in combination with Ad.*mda-7* induction of growth suppression and apoptosis with production of MDA-7/IL-24 protein was evident [78,79]. Though a small number of cells received both agents, the combination treatment resulted in the killing of the majority of the pancreatic cancer cells, for the first time supporting the concept of a “bystander” antitumor effect.

Chada et al. studied the ‘bystander’ activity of MDA-7/IL-24 in melanoma cells where the glycosylated MDA-7/IL-24 produced cell death in a dose-dependent manner, which was mediated through the IL-24 receptors [82]. MDA-7/IL-24 protein induced phosphorylation and nuclear translocation of STAT3 in melanoma cells via both type 1 and type 2 IL-20R and induced dose-dependent cell death in melanoma tumor cells. The effect of MDA-7/IL-24 receptor engagement resulted in the up-regulation of BAX and subsequent apoptosis induction mediated by STAT3-independent signaling. In normal cells, MDA-7/IL-24 can bind to its cognate receptors and induce phosphorylation of STAT3, without cytotoxic sequelae. This study defined a tumor-selective cytotoxic “bystander” role for secreted MDA-7 protein and identified a novel receptor-mediated, STAT3-independent, and PKR-independent death pathway [82]. These results support the hypothesis that in specific contexts and cell types, tumor suppression with *mda-7/IL-24* can be promoted by a subset of cancerous cells producing and secreting MDA-7/IL-24 that suppress adjacent tumor cells, inhibiting survival and tumor development, i.e., “bystander” anti-cancer activity [75,76,77,82].

Su et al., showed that when the cancer cells cocultured with MDA-7/IL-24 secreted from Ad.mda-7-infecetd normal cells, it resulted in the suppression of their growth, invasion and induction of apoptosis [75]. Additionally, the combination of secreted MDA-7/IL-24 and radiation induced a potent “bystander” antitumor effect in both sensitive and resistant prostate cancer cells. Sauane et al. documented that recombinant MDA-7/IL-24 protein could robustly induce expression of endogenous *mda-7/IL-24*, which induced the signaling events necessary for “bystander” antitumor action [76]. This elegant study showed that blocking endogenous *mda-7/IL-24* by siRNA inhibited exogenous MDA-7/IL-24-mediated apoptosis. Mechanistically it was shown that MDA-7/IL-24 protein induced its own mRNA stabilization without activating the promoter and this posttranslational modification depended on de novo protein synthesis [76].

Further confirmatory evidence of MDA-7/IL-24′s “bystander” activity emerged from in vivo animal studies [59,83,84]. In these studies, tumor cells were injected to form tumors in both flanks of nude mice and only the tumor on one side received treatment. Interestingly, a decrease in tumor size was evident not only in the treated tumor but also in the untreated tumor. The inhibitory action on distant tumors was attributed at least in part to the direct “bystander” antitumor activity of the secreted MDA-7/IL-24 which, upon receptor engagement located in the untreated/distant tumors, was able to elicit the signaling events and antitumor response.

### 4.5. Immunogenic Cell Death

MDA-7/IL-24 is expressed in various tissues of the immune system including the spleen, peripheral blood leukocytes (PBL), thymus, and normal melanocytes and functions as a cytokine [6]. MDA-7/IL-24 was shown to play important roles in infectious diseases, wound healing and autoimmune diseases [6,85]. As an anti-cancer agent, MDA-7/IL-24 controls multiple types of tumors, by inducing apoptosis and toxic autophagy, anti-invasion, anti-angiogenesis, sensitizing cancer cells to radiation therapy and chemotherapy [6,7,16,17,18,19,20,21,55,65]. Recent studies also document an immune modulating role of MDA-7/IL-24 as an alternate (or likely additive) mechanism mediating antitumor effects. Caudell et al. demonstrated that treatment of peripheral blood mononuclear cells (PBMC) with MDA-7/IL-24 protein potently induced secretion of several immune modulatory cytokines including IL-6, TNF-α, IFN-γ, IL-1β, and GM-CSF, indicating that MDA-7/IL-24 can function as a pro-Th1 cytokine [64,86]. The same research group also reported that the treatment of melanoma cells with Ad.*mda-7* induced secretion of IL-6 and IFN-γ [64].

Since its discovery, most MDA-7/IL-24-related in vivo studies were performed in immune deficient nude mice xenograft models, hence the function of MDA-7/IL-24 as an immune modulator in vivo in an intact immune-competent setting required further evaluation. Using a syngeneic murine model of fibrosarcoma (UV2237m) Miyahara et al. showed that adenoviral-mediated *mda-7/IL-24* transfer induced anticancer immunity [87]. They found immunocompetent mice injected with Ad.mda-7-transduced UV2237m failed to develop any tumors and when these tumor-free mice were challenged with parental tumor cells, no tumor growth was apparent, suggesting a potential vaccine effect of MDA-7/IL-24 [87]. Ma et al. used a murine model of colon cancer with an intact immune system and reported that MDA-7/IL-24 could inhibit colon cancer progression by exerting immune stimulatory effects [88]. Specifically, they observed that MDA-7/IL-24 promoted IFN-γ secretion from CD4 + and CD8 + T cells and enhanced the cytotoxicity of CD8 + T cells in vivo. The studies by Gao et al. investigated the therapeutic efficacy of Ad.*mda-7* in combination with Ad.*sgrp170* (grp170, an ER resident chaperone) in a TRAMP-C2 prostate cancer model [89]. The study found that the combination treatment of MDA-7/IL-24 and grp170 was more efficient in inhibiting prostate tumor growth as compared to either agent alone. The combined administration of secretable grp170 and MDA-7/IL-24 significantly enhanced the antigen-specific CD8 + T-cell frequency and tumor-specific cytolytic activity, which was further supported by an in vivo antibody depletion study [89]. However, CD8 + depletion did not completely nullify the anticancer effects mediated by the combined therapies, indicating the involvement of other immune effector cells. To further comprehend the role of MDA-7/IL-24 in vivo and elucidate the immune-modulating role in syngeneic breast cancer models with an intact immune system, Menezes et al. performed in vivo experiments using several transgenic models [90]. The results documented that MDA-7/IL-24 expression could delay tumor onset in MMTV-MDA-7/MMTV-Erbb2 compound transgenic mice, and could also suppress tumor growth and exhibit “bystander” antitumor effects in MMTV-PyMT mice. Interestingly, the investigators found that the intra-tumoral injection of Ad.5-*CTV* (replication competent cancer-selective adenovirus expressing MDA-7/IL-24; a cancer terminator virus) resulted in increased CD8 + T cell infiltration and a marked increase in IFN-γ expression in MMTV-PyMT transgenic mice [90]. The enhanced immune activation was observed in both MDA-7/IL-24-treated as well as non-treated tumors, suggesting that a systemic immune response was induced by MDA-7/IL-24 in animals with an intact immune system.

## 5. MDA-7/IL-24 as a Single Therapeutic

Published studies over the last 20 years have validated the finding that forced expression of MDA-7/IL-24, either by transfection of tumor cells with an *mda-7/IL-24* cDNA containing plasmid, or by use of a recombinant adenovirus (Ad.*mda-7*) expressing *mda-7/IL-24,* significantly inhibits the growth of a diverse spectrum of cancer cells, both in vitro and in vivo in animal models as a single therapeutic [4,5,6,7,16,17,18,19,20,21,55,65]. Although *mda-7/IL-24* has consistently proven efficient in inducing antitumor effects in multiple types of cancers when delivered by Ads. or plasmid transfection, there is always room for better and more efficient and specific delivery both in vitro and in vivo. Accordingly, studies have focused on improving the delivery of *mda-7/IL-24* to tumor cells and defining ways to enhance anti-cancer effects.

### 5.1. Virus-Mediated Gene Delivery

The majority of studies have utilized a replication incompetent Ad expressing *mda-7/IL-24* (Ad.*mda-7*) for delivering MDA-7/IL-24 to cancer cells. Several modifications have been explored to improve selective delivery to cancer cells, which we will briefly discuss in this section.

#### 5.1.1. Tropism Modification

The Ad.*mda-7* virus tested in most studies employ serotype 5 Ads (Ad.5), which utilize coxsackie-adenovirus receptors (CARs) on the cell surface as the prime means of infecting cells and the efficiency of infection is dependent upon the level of CAR expression on target cells [91]. To mitigate this issue related to poor infectivity of low-CAR cells, several research groups, including ours, used “tropism modification” techniques which incorporate type 3 Ad sequences within the Ad type 5 virus knob. This modification (Ad.5/3) promotes high infectivity in low as well as high CAR expressing tumor cells showing equal efficacy when compared with the original Ad.5 [58,92,93]. The improved transgene delivery and efficacy of an Ad.5/3 recombinant virus expressing *mda-7/IL-24*, has been demonstrated in prostate cancer, glioma, colorectal, and renal cancer [58,92,93,94].

#### 5.1.2. Conditionally Replication Competent Ad (CRCA)

A common obstacle often encountered when using Ad-mediated gene therapy is the stimulation of immune responses resulting in viral neutralization and clearance following multiple Ad injection. Considering these drawbacks, CRCAs were engineered that induce oncolysis by cancer-specific replication and were evaluated in clinical trials [8,9,10,95]. To improve the therapeutic efficacy and cancer-specific delivery of MDA-7/IL-24, Sarkar et al. created a bipartite CRCA expressing *mda-7/IL-24* [10,83,84,96], in which E1A and E1B (replication component) expression is regulated by a cancer-selective minimal active region of the promoter of progression elevated gene-3 (PEG-3), which functions selectively in diverse cancer cells with limited activity in normal cells [97,98]. This virus, also called a cancer terminator virus (CTV), induces cancer-specific replication and a second CMV promoter in the virus promotes MDA-7/IL-24 expression uniquely in cancer cells as a consequence of virus replication [83,84,96]. The robust and superior cancer selective activity of the CTV (developed both in serotype 5 ad 5/3 Ads) was validated in diverse cancers including prostate, glioma, melanoma, breast, pancreatic and neuroblastoma both in vitro and in vivo using established cancer cell lines, patient-derived cancer cells, human tumor xenografts in nude mice, murine tumor xenografts in immune competent mice and genetically engineered transgenic mouse models of several cancers [8,9,10,83,84,90,96,99,100,101]. Another CRCA (ZD55-IL-24) was created to deliver mda-7/IL-24 using the ZD55 vector, in which the adenoviral E1B 55-kDa gene was deleted to control replication in cancer cells with p53 dysfunction. The efficacy of the virus was tested in colorectal cancer and infection of colorectal cancers with ZD55-IL-24 showed a greater antitumor effect than observed with Ad.*mda-7/IL-24* or ONYX-015 (a virus that preferentially replicates in cells with defective p53) [102]. The efficacy of ZD55-IL-24 was also confirmed in other cancer indications, including B-lymphoblastic leukemia, leukemia, breast cancer and melanoma. [103,104,105,106]. These profound results with CTV and other CRCAs expressing *mda-7/IL-24* support their use as a potential therapeutic for diverse cancer indications.

#### 5.1.3. Ultrasound-Targeted Microbubble-Destruction (UTMD): A Strategy for Targeted Delivery of Therapeutic Agents

Major hurdles limiting efficient Ad gene therapy in the clinic include the host’s antiviral immune responses, which can limit multiple administration, and viral entrapment in the liver, when delivered systemically using the intravenous route [107]. Ultrasound (US) contrast agents (microbubbles) have recently emerged as a potential agent for effectively delivering therapeutics to target tissues [108,109,110]. Microbubbles (MB) containing therapeutics (Ads/small molecule drugs/chemotherapeutic drugs) can be injected in peripheral veins, and when sonoporated using ultrasound causing the focal release of entrapped materials in the targeted region (UTMD; ultrasound targeted microbubble destruction). Greco et al. showed that Ad.*mda-7* complexed in MBs when injected and sonoporated, efficiently reduced tumor size in tumor-bearing mice [111]. Dash et al. tested the efficacy of Ad.5/3-mda-7 and BI-97C1, an MCL-1 inhibiting small molecule, in a spontaneous model of prostate cancer using the Hi-Myc mouse [112]. The Hi-Myc mice received intravenous injections of complement-treated MBs containing Ad.5/3-mda-7 virus, followed by sonoporation in the prostatic region. This study revealed that the sizes of the prostates of Hi-Myc mice treated with the combination of Ad.5/3-mda-7 and the Apogossypol derivative BI-97C1 were significantly smaller compared with treatment with either agent alone or the vehicle control [112,113]. A study by Sarkar et al. further refined the UTMD approach using a decorated MB (D-MBs), in which biotinylated anti-V-CAM-1 was complexed with streptavidin microbubbles resulting in D-MBs [114]. These D-MBs accumulated in the tumor vasculature and, after sonoporation in the prostate region in the Hi-Myc mice, the therapeutic viruses were released. These studies support the use of MB/D-MBs and UTMD as a novel systemic delivery modality to deliver viruses (such as Ad.*mda-7/CTV*) to internal tumors (such as prostate tumors in Hi-Myc mice), resulting in profound anti-cancer activity.

### 5.2. T Cells Expressing MDA-7/IL-24

One recent study by Liu et al. evaluated the therapeutic activity of tumor-reactive or antigen-specific T cells genetically engineered to express human MDA-7/IL-24 [11]. Current cellular immunotherapies that use tumor-infiltrating lymphocytes or engineered chimeric antigen receptor (CAR)-T cells, have shown limited efficacy in the treatment of solid cancers, which are known to be highly heterogenous and display variable antigen expression [115,116]. Liu et al. used multiple clinically relevant mouse syngeneic tumor models, including a transgenic spontaneous prostate cancer model. MDA-7/IL-24-expressing T cells (T-MDA-7) were superior to unmodified T cells in suppressing primary mouse prostate cancer and melanoma as well as inhibiting cancer metastases [11]. Hi-Myc transgenic mice that spontaneously develop prostate cancer were also used to evaluate the therapeutic potency of human MDA-7/IL-24-producing T cells. Different cohorts of Hi-Myc mice were treated with tumor-sensitized T cells that were engineered to produce human MDA-7/IL-24. Examination of the prostates after euthanizing animals at 6 months showed that T cell-MDA-7 therapy was more effective than mock engineered T cells at inhibiting prostate cancer progression. Administration of MDA-7/IL-24-producing T cells was associated with elevation of mRNA levels of human MDA-7/IL-24, and mouse TNF-α, and IFN-γ in prostate tissues, as a result of T-cell trafficking to the tumor sites and homing to lymphoid organs [11]. Histological analysis of prostate tissues showed that treatment with MDA-7/IL-24-producing T cells significantly reduced progression of prostate cancer. Altogether, these findings suggest that MDA-7/IL-24-engineered T cells exhibit superior anticancer activity by offsetting multiple immune limiting factors in the tumor microenvironment and targeting cancer cells beyond an antigen-specific fashion [11].

### 5.3. Recombinant MDA-7/IL-24 Protein

Initial studies by Sauane et al., indicated that recombinant MDA-7/IL-24 protein (His-MDA-7) induced endogenous mda-7/IL-24 expression, which then instigated signaling events necessary for “bystander” antitumor effects [76]. Using recombinant His-MDA-7 protein, Dash et al. observed that MDA-7/IL-24, after binding to its cognate receptors, induced intracellular SARI (suppressor of AP-1, regulated by IFN) expression [23]. Activation of cognate IL-20/IL-22 receptors by MDA-7/IL-24 resulted in phosphorylation of p38 MAPK (mitogen activated protein kinase) signaling pathways, which in turn activated GADD (growth arrest and DNA damage inducible) genes, subsequently leading to apoptosis. One recent study by Pradhan et al. suggests that recombinant His-MDA-7 downregulated miR-221, exclusively in IL-20/IL-22 receptor positive cancer cells [59]. When the complete sets of cognate receptors were reconstructed in receptor negative cells, these cells then displayed sensitivity to His MDA-7 treatment that resulted in the downregulation of miR-221. Further studies by our group elucidated the underlying mechanism of regulation of miRNAs by MDA-7/IL-24 [39]. We found that MDA-7/IL-24 (either by Ad-mediated delivery or with pure recombinant protein) downregulated DICER in a reactive oxygen species-dependent manner and the overexpression of DICER partially rescued MDA-7/IL-24-mediated cell death in cancer cells. Additionally, we observed that MDA-7/IL-24-mediated DICER regulation was mediated by MITF (transcription factor melanogenesis-associated transcription factor).

### 5.4. Nanoparticle-Mediated Delivery

Nanoparticle-mediated gene delivery is an alternative methodology that is suitable for systemic applications in the clinic [117,118]. Recently, nanoparticles have emerged as a powerful tool to deliver therapeutic payloads to disease sites, which include nucleic acids, pure proteins, virus-like particles, small molecule drugs, chemotherapeutic agents, etc. Nanoparticles can also be delivered as biodegradable microspheres, bioresorbable solid implants, injectable gels, and drug-eluting coatings. Ramesh and colleagues evaluated nanoparticle-mediated delivery of *mda-7/IL-24* in primary and disseminated lung cancer [119]. The results of the study revealed that DOTAP:chol (DOTAP:Cholesterol) nanoparticles efficiently deliver *mda-7/IL-24* to human lung tumor xenografts, resulting in growth suppression in both primary and metastatic tumors and they also inhibited tumor angiogenesis. Additionally, they found that DOTAP:Chol–*mda-7/IL-24* nanoparticles also inhibited growth of murine tumors in syngeneic mouse cancer models [119]. Future studies using the nanoparticle technology to deliver MDA-7/IL-24 are definitely warranted.

## 6. Combination Effects of MDA-7/IL-24 with Other Therapeutic Agents

Cancer is a complex, heterogenous, multi-factorial disease that occurs as a result of dysregulated epigenetic and genetic events controlling critical signaling mechanisms involved in cell growth, resistance to apoptosis and other physiological processes [120]. Cancer patients frequently develop resistance to a targeted therapy due to the activation of compensatory pathways that cancer cells exploit to survive. As such, combination approaches with multiple targeted agents may provide superior therapeutic benefit as compared to a single agent alone, which continues to be observed in pre-clinical studies and in the clinic. Although MDA-7/IL-24 is effective when delivered as a single therapy, antitumor activity is further augmented in a synergistic manner when this cytokine is combined with other therapies, including, chemotherapy, radiation, antibody-based therapies, small molecule therapies, and immunotherapies (summarized in Table 1) [6,7,16,17,18,19,20,21,55,65].

MDA-7/IL-24 has the unique ability to efficiently kill almost all types of cancer cells, but pancreatic cancer cells having K-Ras mutations, show an inherent resistance to MDA-7/IL-24 treatment [78,79,133]. This type of resistance can be successfully reversed by combining *mda-7/IL-24* with antisense K-Ras treatment or inhibition of K-Ras-downstream ERK1/2 signaling [78]. Subsequent studies by Lebedeva et al. reported that a dietary monoterpene, perillyl alcohol (POH), at low doses significantly improved the efficacy of Ad.mda-7 therapy in resistant pancreatic carcinoma cells [134,135]. The mechanism of the synergistic effects observed in combination therapy involving MDA-7/IL-24 and POH was attributed to the generation of reactive oxygen species (ROS) blocking of which with the ROS inhibitor (N Acetyl l Cysteine-NAC) significantly mitigated the growth inhibitory effects induced by the combination treatment. Other ROS generating therapeutic agents, including arsenic trioxide, 4-hydroxyphenyl-retinamide (4-HPR), have also been shown to augment the therapeutic effect of MDA-7/IL-24 in cancers of the pancreas and kidney [136,137].

Radiotherapy is a standard of care (SOC) treatment option for many cancers. Several research groups have established that MDA-7/IL-24 can radiosensitize a diverse array of cancer cell lines both in vitro and in vivo [21,65,67,123,138,139,140,141,142,143]. The underlying mechanisms involved in this radiosensitization effect include generation of ROS and ceramide and signaling pathways such as activation of c-Jun NH2-terminal kinase (JNK), p38MAPK-GADDs. Non-small cell lung cancer (NSCLC) cells are radiosensitized by Ad.*mda-7* via JNK1/2 signaling [67]. Yacoub et al. demonstrated that in glioma, Ad.*mda-7* caused radiosensitization in vitro and in vivo, documenting that this sensitization was dependent on JNK1/2/3 activation [139,144]. The radiosensitization effect of MDA-7/IL-24 has also been reported in ovarian, prostate, breast and nasopharyngeal cancers [123,141,142,145].

Ceramide is an important component in MDA7/IL-24-mediated induction of cancer cell-specific apoptosis. Accordingly, Carmofur (an acid ceramidase inhibitor) promotes ceramidase accumulation in cells after MDA-7/IL-24 treatment inducing cancer-specific apoptosis [56]. In combination with trastuzumab/herceptin, Ad.*mda-7* has also been reported to decrease the tumor size in pre-clinical animal models containing breast cancer cells overexpressing Her-2/neu receptors as a result of inhibition of the Wnt signaling pathway, a promoter of cancer progression [122]. In another study, Ad.*mda-7* was used in combination with bevacizumab in a lung cancer model and the combination treatment significantly enhanced apoptosis induction in vitro. When used in a lung cancer xenograft model, tumors receiving Ad.*mda-7* plus bevacizumab showed complete tumor regression at the completion of the study [121]. The combination effect of MDA-7/IL-24 with temozolomide, an alkylating agent, was investigated in human melanoma and glioma cells [146,147]. In both cancer contexts, addition of MDA-7/IL-24 helps overcome resistance to temozolomide. In temozolomide-resistant melanoma cells, MDA-7/IL-24 caused inhibition of O6-methylguanine-DNA methyltransferase (MGMT) resulting in enhanced temozolomide-induced cell killing [146]. Emdad et al. investigated the efficacy of the combination of Ad.mda-7 with a selective EGFR inhibitor, gefitinib, in NSCLC [132]. The investigators found that combination treatment resulted in enhanced apoptotic cell death in the treated cells by increasing the expression of a downstream effector molecule, RNA-activated protein kinase. This observation was further extended by Gupta et al. who demonstrated that combination treatment of either GST-MDA-7 or GST-M4 (a truncated version of MDA-7/IL-24) and Tarceva (erlotinib) at sub-optimal doses synergistically inhibited non-small cell lung carcinoma (NSCLC) cell growth and enhanced apoptosis as compared to either agent alone [148]. *mda-7/IL-24* incorporated in ZD55-IL-24 (an oncolytic adenovirus) was used in combination with cisplatin in a panel of cancer cells and the combination treatment markedly enhanced the cytotoxicity and apoptosis in all of the cancer cells tested [149]. Intriguingly, no harmful toxic effect was evident in the normal cellular counterparts. MDA-7/IL-24 also acted synergistically in colorectal cancer and prostate cancer cells when combined with the Apogossypol derivatives, BI-97C1 and BI-97D6, which are pharmacological inhibitors of Mcl-1 [94,112,114]. Combined treatment with Ad.5/3-*mda-7* and BI-97C1 significantly enhanced toxicity in human prostate cancer cells, inhibited the growth of prostate cancer xenografts in vivo and controlled cancer development in a transgenic immune-competent mouse model of prostate cancer (Hi-Myc model) [112]. The combination treatment of Ad.5/3-*mda-7* and BI-97C1 caused up-regulation of NOXA, Bax, Bak and Bim, resulting in toxic autophagy and apoptosis induction. Sorafenib tosylate (a potent multi kinase inhibitor and a clinically proven FDA approved drug in hepatocellular carcinoma) treatment in combination with MDA-7/IL-24 has also been reported to kill renal carcinoma cells in vivo in animal models resulting in prolonged survival [150]. MDA7/IL-24 induces cell death by enhancing endoplasmic reticulum (ER) stress and also leads to an increase in DNA acetylation. Consequently, HDAC inhibitors have also been reported to work synergistically with MDA7/IL-24 in killing renal carcinoma cells and glioblastoma [42,43]. The future of MDA-7/IL-24 combination treatments is rife with opportunities that need to be explored further, based on its multidimensional therapeutic ability and cancer cell-specific killing properties. These traits establish MDA-7/IL-24 as one of the most promising therapeutic cytokines in the cancer research field.

## 7. Phase I Clinical Trial of MDA-7/IL-24 (Ad.5-*mda-7*:INGN-241)

**Figure 7:**
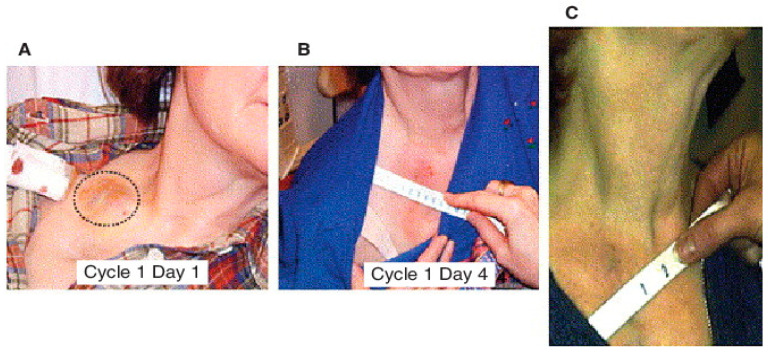
Objective clinical response to INGN 241 (Ad.5-*mda-7*) in cohort 8 patient with metastatic melanoma (patient 83). Injected lesion was on right clavicle (dashed circle in (**A**)). (**B**) by day 4, the region is inflamed. (**C**) At the end of cycle 1 (day 30), the lesion has completely regressed. This patient was alive > 600 days post INGN 241 treatment. Reprinted by permission from Lebedeva et al. 2007 [65].

The efficacy of MDA-7/IL-24 as a cancer therapeutic has been established in pre-clinical studies using multiple tumor models, including nude mice, syngeneic mice and transgenic animals [6,7,16,17,18,19,20,21,55,65]. Based on profound and selective antitumor activity in vitro and in animal models, a replication incompetent type 5 Ads expressing MDA-7/IL-24 (Ad.5-*mda-7*; INGN-241) was tested in a Phase I clinical trial in patients with advanced cancers, including melanomas and carcinomas [17,21,65,151,152,153,154,155,156]. The virus was delivered via intra-tumoral injections to 28 patients diagnosed with melanoma, colorectal cancer, breast cancer, squamous cell carcinoma of the head and neck (SCCHN), lymphoma, adenocarcinoma, non-small cell lung cancer, hepatoma, sarcoma and carcinomas of the bladder adrenal gland and parotid gland. The patients enrolled in this clinical study received prior treatment with radiation, chemotherapies and/or surgery. A dose-escalation approach was employed, and patients received 2 × 10^10^ to 2 × 10^12^ viral particles INGN-241 (Ad.5*-mda-7*) injected into the central region of the target tumor. At different time points (24 h to 30 days post injection), the tumors were resected and evaluated for vector-specific DNA and RNA, transgenic MDA-7/IL-24 expression, and biological effects. DNA and RT-PCR analysis revealed a successful gene transfer in 100% of patients that received the INGN-241 (Ad.5-*mda-7*) injection. The tumor lesions received INGN 241 (Ad.5-*mda-7*) injections showed high levels of MDA-7/IL-24 protein expression, which correlated well with the apoptotic activity as evidenced by TUNEL assay. Tumor sections were also evaluated for the expression of β-catenin, iNOS (inducible nitric oxide synthase) and CD31 (angiogenesis marker), all of which were reduced post INGN-241 (Ad.5-*mda-7*) treatment. Patients who received multiple injections of INGN-241 (Ad.5-*mda-7*) did not show any sign of overt toxicity and a durable clinical response rate (~44%) was observed in a subset of patients. The most promising response was observed in a patient with metastatic melanoma with more than 10 distinct lesions. Initially, a lesion in the supraclavicular node (2 × 2 cm) was injected with INGN-241 (Ad.5-*mda-*7) and after the sixth injection, a gradual decrease in the lesion size was evident which continued over the following 2 weeks with no clinical evidence of the disease (Figure 7). A lesion on the dorsum of the right hand (1.8 × 2.3 cm) was treated next and with five injections, there was an 84% reduction in the lesion area with microscopic lymphoplasmacytic infiltrations and extensive coagulative necrosis. A third lesion in the anterior right thigh (3.5 × 3 cm) showed 35% reduction [152]. This patient survived more that 600 days post INGN 241 (Ad.5-*mda-7*) treatment.

The “bystander” activity of MDA-7/IL-24 was also demonstrated in this clinical trial [151,152]. As an example, a single injection of INGN 241 (Ad.5-*mda-7*) resulted in the transduction of only 10% to 30% of the tumor mass, however, intriguingly, 70% of the tumor cells exhibited signs of apoptosis, supporting the antitumor ‘bystander’ effect observed in vitro and in vivo in animal models. The immune modulating role of MDA-7/IL-24 observed in vitro was also recapitulated in these patients following INGN-241 (Ad.5-*mda-7*) injection, as evidenced by a transient increase in circulating cytokines, such as IL-6, IL-10 and TNF-α [150,151]. At day 15 after injection, the majority of patients also showed marked increases in CD3^+^ and CD8^+^ T cells, suggesting that INGN-241 (Ad.5-*mda-7*) may be associated with a Th1 response [151]. These initial clinical studies evaluated the safety profile, pharmacodynamics, pharmacokinetics of vector-specific DNA, mRNA, MDA-7/IL-24 protein distribution and its biological effects, both locally and systemically. In all patients, multiple intra-tumoral injections with INGN 241 (Ad.5-*mda-7*) were found to be safe, and any adverse events were relatively mild, which is very promising and paves a path to develop future clinical trials with improved next generation versions of MDA-7/IL-24 therapeutic, alone and, more profoundly, in combination with other treatment modalities.

## 8. Conclusions and Future Prospective

**Figure 8:**
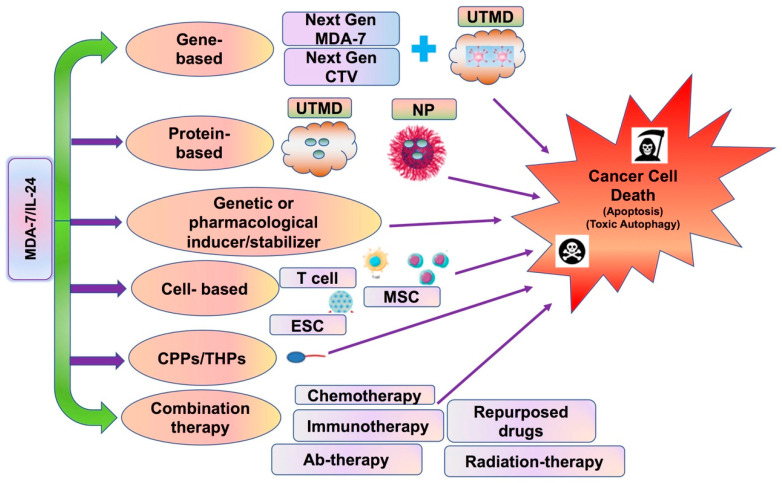
**Strategies to improve MDA-7/IL-24 as a therapeutic for cancer**. This figure provides various strategies that offer promise of enhancing the ability to use MDA-7/IL-24 as an effective therapy for multiple cancer subtypes. These approaches that include MDA-7/IL-24 include gene-based, protein-based, use of genetic or Pharmacological inducers/stabilizers, cell-based, cell-penetrating peptides or tumor homing peptides, antibody-based and combination therapies (chemotherapy, immunotherapy, repurposed drugs, Ab-therapy, small molecule therapy, radiation therapy, etc.). Abbreviations used in this figure: Next Gen MDA-7: Next Generation MDA-7; CTV: cancer terminator virus; Next Gen CTV: Next Generation cancer terminator virus; UTMD: ultrasound targeted microbubble destruction; NP: nanoparticle; ESC: embryonic stem cells; MSC: mesenchymal stem cells; CPP: cell penetrating peptides; THP: tumor homing peptides; Ab-therapy: antibody-based therapy.

MDA-7/IL-24, a multifunctional cytokine and member of the IL-10 gene family, displayed profound anti-cancer activities in pre-clinical studies and in Phase I clinical trials in patients with advanced cancers. Several research laboratories in the USA and globally have studied the biological functions and molecular mechanisms of MDA-7/IL-24 for more than two decades, which has enriched our understanding about the multiple functions of this potent cancer therapeutic. Although the results of the Phase I clinical trial were promising, it is obvious that further improvements in many areas indicated below are required to maximize the enhanced therapeutic benefits of MDA-7/IL-24 in patients. The cancer cell-specific killing ability of MDA-7/IL-24 cytokine is still an unresolved mystery, which may involve differences in altered metabolism, inherent biochemical and oxidative stress conditions between normal and cancer cells. Specific areas of research that may provide tangible benefits in improving MDA-7/IL-24 therapeutic properties are shown schematically in Figure 8. Strategies to achieve this objective include: (1) Genetically engineering newer versions of MDA-7/IL-24, rendering it more stable with increased secretion (which should enhance further “bystander” anti-cancer activity). (2) Developing a newer next generation of CRCAs (including *CTVs*) containing second next generation MDA-7/IL-24 controlled by more specific and robust cancer-selective promoters. (3) Identification of new molecules/targets/miRNAs/long non-coding RNAs that can enhance or stabilize the MDA-7/IL-24 protein. (4) Development of effective systemic delivery approaches for MDA-7/IL-24. Ad-mediated delivery of MDA-7/IL-24/CTV is very efficient in inhibiting tumor growth when delivered intratumorally, however, systemic administration of these therapeutic viruses may be necessary to achieve maximum benefit and for better disease control. Since MDA-7/IL-24 can initiate autocrine/paracrine production of MDA-7/IL-24, the systemic administration of this purified protein may cause serious adverse effects to humans, such as nonspecific inflammation and autoimmunity, which would restrict its therapeutic application as a pure systemically administered protein. In this context, using UTMD or nanoparticles to deliver MDA-7/IL-24-based therapies, may obviate these issues of potential “cytokine storm” effects that might occur upon systemic delivery of MDA-7/IL-24 as a pure protein. (5) Use of cell-based vehicles (such as T cells, embryonic stem cells, mesenchymal stem cells, etc.) for targeted delivery of MDA-7/IL-24 is also an area of active research that would be very useful for developing novel MDA-7/IL-24-based therapies. (6) Some recent studies are using a fusion engineering approach where MDA-7/IL-24 is fused with cell penetrating peptides (CPPs) or tumor homing peptides [157,158] to target it to neighboring tumor sites to enhance its therapeutic efficiency. And finally: (7) Discovering novel combination therapies with other therapeutics (including chemotherapy, antibody-based therapy, immunotherapy, repurposed FDA-approved drug, etc.) that would enhance MDA-7/IL-24-mediated anticancer effects. As we have tried to capture, MDA-7/IL-24 has significant potential as a therapeutic for multiple primary and metastatic cancers and exploiting these properties has significant potential to lead to effective and enduring therapies for cancer.

## Figures and Tables

**Table 1:** Combinatorial therapy of combining mda-7/IL-24 with other therapeutic modalities.

COMBINATION TREATMENT	CANCER TYPE	MECHANISM	REF
Ad.5mda-7 + bevacizumab	Lung tumor xenograft	Treated lung tumor cells showed lower VEGF ligand-receptor binding, lower cell survival, significant growth arrest and apoptosis.	[121]
Ad.5/3mda-7 + HDAC inhibitor	Renal cell carcinoma	This combination led to activation of CD95, dihydro-ceramide/ROS/Ca2 + generation and ER stress.	[43]
Ad5.mda-7 + trastuzumab (Herceptin)	Breast cancer	Inhibited the β-catenin and AKT pathway in HER-2/neu overexpressing breast cancer cells.	[122]
Ad5.mda-7 + radiotherapy	Breast cancer	Mda-7 expressing cells showed synergistic cytotoxicity and apoptosis due to decreased Bcl-2 expression and Bax upregulation.	[123]
Ad5.mda-7 + cox-2 inhibitor (celecoxib)	Breast cancer	Mda-7 treatment downregulated AKT and simultaneously inhibited Cox-2 expression, promoting apoptosis.	[124]
Ad.5/3.mda-7 + cisplatin/paclitaxel	Ovarian cancer	Combination of paclitaxel significantly enhanced (additive effect) the tumor cell killing by Ad5/3.mda-7 + cisplatin treatment.	[125]
Ad5.mda-7 + sabutoclax (BI-97C1)	Prostate cancer	Sabutoclax inhibited mcl-1 and synergized with mda-7, preventing tumor growth, angiogenesis and regulating immune responses.	[112]
F5/35-Zd55-IL-24 + temozolomide	Melanoma	F5/35-zd55-IL-24 and TMZ increased the level of pro-apoptotic proteins and decreased anti-apoptotic proteins.	[126]
IL-24 + cisplatin	Cervical cancer	IL-24 (mda-7) enhanced the tumor chemosensitivity to cisplatin by downregulating the VEGF, VEGF-c and PDGF-b expression.	[127]
Zd55-IL-24 + dichloroacetate	Liver cancer	This combination treatment promoted translocation of Bax from the cytoplasm to mitochondria and promoted apoptosis, without altering bcl-2 expression.	[128]
IL-24 + Erlotinib	Melanoma	IL-24 (mda-7) sensitized melanoma cells to Erlotinib by modulating apaf-1 and AKT pathways.	[129]
Tat-IL-24-kdel + survivin inhibitor (ym155)	Melanoma	Inhibition of survivin promoted the apoptosis promoting efficiency of tat-IL-24-kdel in melanoma cells.	[130]
vv-IL-24 + luteolin	Hepatic cancer	Luteolin promoted vv-IL-24 gene expression in liver cancer cells using in vitro and in vivo experiments.	[131]
Ad.mda-7 + gefitinib	Non-small cell lung cancer	This combination inhibited p-EGFR, p-ERK and p-AKT levels in NSCLC cells.	[132]

## Data Availability

Not applicable.
